# A Novel Rat Model of Blast-Induced Traumatic Brain Injury Simulating Different Damage Degree: Implications for Morphological, Neurological, and Biomarker Changes

**DOI:** 10.3389/fncel.2015.00168

**Published:** 2015-05-01

**Authors:** Mengdong Liu, Chi Zhang, Wenbo Liu, Peng Luo, Lei Zhang, Yuan Wang, Zhanjiang Wang, Zhou Fei

**Affiliations:** ^1^Department of Neurosurgery, Xijing Hospital, Fourth Military Medical University, Xi’an, China; ^2^Northwest Institute of Nuclear Technology, Xi’an, China

**Keywords:** blast-induced traumatic brain injury, animal model, biomarker, inflammation, metabolic change

## Abstract

In current military conflicts and civilian terrorism, blast-induced traumatic brain injury (bTBI) is the primary cause of neurotrauma. However, the effects and mechanisms of bTBI are poorly understood. Although previous researchers have made significant contributions to establishing animal models for the simulation of bTBI, the precision and controllability of blast-induced injury in animal models must be improved. Therefore, we established a novel rat model to simulate blast-wave injury to the brain. To simulate different extents of bTBI injury, the animals were divided into moderate and severe injury groups. The miniature spherical explosives (pentaerythritol tetranitrate) used in each group were of different sizes (2.5 mm diameter in the moderate injury group and 3.0 mm diameter in the severe injury group). A specially designed apparatus was able to precisely adjust the positions of the miniature explosives and create eight rats with bTBI simultaneously, using a single electric detonator. Neurological functions, gross pathologies, histopathological changes and the expression levels of various biomarkers were examined after the explosion. Compared with the moderate injury group, there were significantly more neurological dysfunctions, cortical contusions, intraparenchymal hemorrhages, cortical expression of S-100β, myelin basic protein, neuron-specific enolase, IL-8, IL-10, inducible nitric oxide synthase, and HIF-1α in the severe injury group. These results demonstrate that we have created a reliable and reproducible bTBI model in rats. This model will be helpful for studying the mechanisms of bTBI and developing strategies for clinical bTBI treatment.

## Introduction

Blast injuries, a type of complex physical trauma resulting from the wide use of explosive devices, have caused large numbers of casualties in military conflicts (Jaffee et al., 2009). Blast-induced traumatic brain injury (bTBI) attracts much attention because it is one of the most serious wounds suffered by warfighters in the battlefield and is responsible for an increasing number of civilian injuries (Frykberg, 2002; de Ceballos et al., 2005; Warden, 2006; Bochicchio et al., 2008). Furthermore, the persistent impairments caused by bTBI in battlefield survivors can also result in psychological symptoms, which may correlate with post-traumatic stress disorder (PTSD) (Belanger et al., 2009; Bolzenius et al., 2014).

Blast-induced traumatic brain injury results from the process of a blast pressure wave and energy acting on the brain (Bhattacharjee, 2008; Ritenour and Baskin, 2008). The extent of bTBI is affected by the parameters of the blast wave, including the peak pressure, duration, and shape of the pulse (Risling and Davidsson, 2012). However, these important parameters can vary depending on the environment and armor conditions. Moreover, other various factors such as penetrating fragments, body acceleration and deceleration, and thermal and chemical injuries further add to the complexity of bTBI (DePalma et al., 2005; Taber et al., 2006; Kluger et al., 2007). All of these factors make it quite difficult to collect enough information from epidemiological data and individual cases to study the mechanisms of bTBI. In the past several decades, various types of experimental animal models have been used to investigate the mechanisms of bTBI, including open field exposure models and blast or shock tube models (Saljo et al., 2000; Long et al., 2010, 2009; Cheng et al., 2010; Alley et al., 2011; Rubovitch et al., 2011). However, the precision and controllability of these models are insufficient. Data obtained from these models are difficult to analyze and compare for the purpose of studying the mechanisms of bTBI. Therefore, it is important to generate well-designed bTBI animal models.

In this study, we describe and validate a novel bTBI model in rats, which precisely positions the area of injury and controls the extent of damage. We believe that this model will be beneficial to study the mechanisms of bTBI. These data can hopefully be used for developing bTBI clinical treatment strategies.

## Materials and Methods

### Ethics statement

All experimental protocols and animal handling procedures were performed in accordance with the National Institutes of Health (NIH) guidelines for the use of experimental animals and approved by the Institutional Animal Care and Use Committee of the Fourth Military Medical University (Permit Number: fmmu-13-0911).

### Animals and experimental design

Male adult Sprague-Dawley rats weighing between 250 and 300 g (Experimental Animal Center of the Fourth Military Medical University, Xi’an, PR China) were randomly divided into two groups: a moderate injury group (70 rats) and a severe injury group (70 rats). All rats were kept five per cage at room temperature under a constant 12-h light/dark cycle. Food and water were available *ad libitum*.

### Blast trauma model

**Figure 1:**
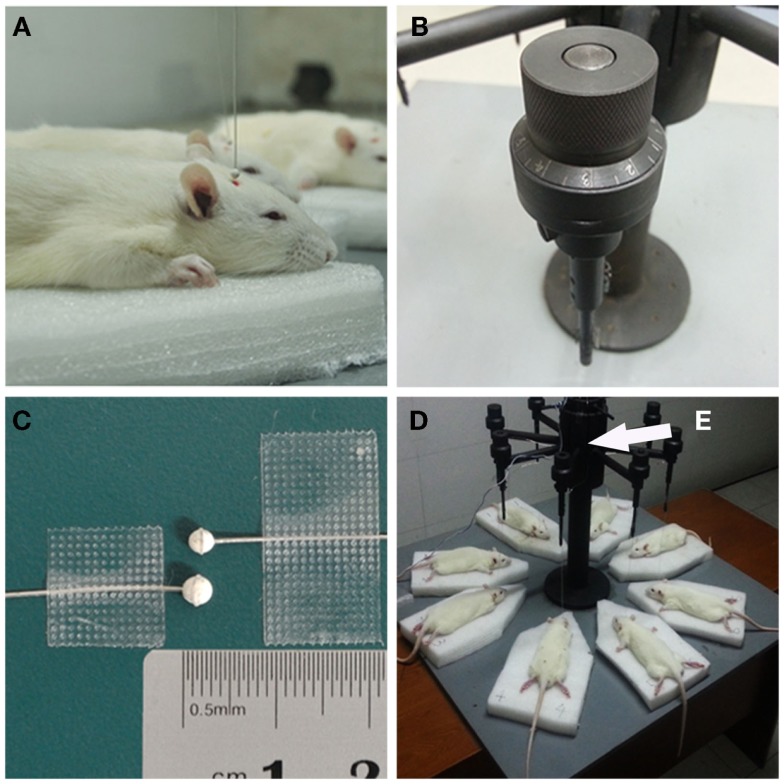
**Details of blast-induced traumatic brain injury (bTBI) model**. **(A)** The distance and relative position between exploder and experimental animals were precisely controlled to ensure the miniature spherical explosives were placed 2.0-mm over the right frontal parietal lobe of the rats (3 mm right to the center point between bregma and lambdoid suture). **(B)** The knob on the apparatus could precisely adjust the position of miniature spherical explosives. **(C)** The standard of 2.5-mm diameter miniature spherical explosive with the explosive equivalent 15.6 mg TNT (above), and 3.0-mm diameter spherical exploder with the explosive equivalent 27.0 mg TNT (below). **(D)** The specially designed apparatus were able to create eight bTBI rats at one time by a single electric detonator. The electric detonator was fixed inside the metal sleeve to isolate the broken pieces **(E)**. The detonator and a silver fuse ignited the miniature spherical explosive, which generated the blast wave. By turning the knob on the top of each miniature spherical explosive holder and moving the sponge board around, the damage position and relative distance between exploders and rats could be precisely and independently adjusted.

Before the explosion, the rats were anesthetized by intraperitoneal (i.p.) injection of chloral hydrate (400 mg/kg). Anesthetized rats were placed on soft sponge boards under the miniature spherical explosives (Figure 1A). The position of the miniature explosive can be precisely adjusted by a knob on the apparatus, which we specially designed (Figure 1B). In our study, the miniature explosives were fixed 2 mm over the scalp and 3 mm right of the center point between the bregma and lambdoid suture. Miniature spherical explosives of 2.5 and 3.0 mm diameter were used in the moderate injury and severe injury groups, respectively (Figure 1C). All miniature spherical explosives were composed of pentaerythritol tetranitrate (PETN) and produced in accordance with these constant parameters; charge density, 1.50 g/cm^3^; detonation velocity, 7500 m/s; detonation pressure, 22 GPa; the TNT equivalents in the 2.5- and 3.0-mm diameter spherical exploders were 15.6 and 27.0 mg, respectively (Northwest Institute of Nuclear Technology, Xi’an, PR China). The specially designed apparatus was able to create eight bTBI rats at one time using a single electric detonator (Figures 1D,E). After triggering the electric detonator, the blast wave was released upon detonation of the miniature spherical explosives by a silver fuse (Video S1 in Supplementary Material). Before explosion, 10 rats from each group were randomly selected as controls, which were treated in the same way but without experiencing an explosion. The injured and uninjured rats were returned to their cages for recovery. The condition of the rats was monitored every 30 min.

### Neurological severity score assessment

The neurological severity score (NSS) was recorded to assess neurological impairments of rats, which included reflexes and motor functions as previously described (Shohami et al., 1996; Zohar et al., 2003; Milman et al., 2005). The animals were reevaluated at various time points (6, 12, 24, 72 h, and 1 week) after explosive injury. Task of assessment was performed by three independent experimenters who were blinded to the animal groups.

### Gross observation and histopathological examination

After explosion, the rats surviving and recovering from anesthesia were sacrificed by cervical dislocationat at different time points (6, 12, 24, 72 h, and 1 week). Control rats were sacrificed after recovering from anesthesia before explosion (pre). Sacrificed rats were initially perfused with 10% formalin, and underwent craniotomy after fixation. Brain tissues were harvested for evaluating the cortical contusion, intraparenchymal hemorrhage, and laceration. Then the tissues were fixed with 10% formalin, dehydrated by graded ethanol, and then embedded in paraffin. The 5 μm-thick slices were cut for HE and Nissl staining to identify injury degree and injury area. The percentage of injured area to whole brain area was evaluated on six Nissl-stained sections by using Image Pro Plus 6.0 software package. In addition, heart, spleen, kidney, lung, and liver were removed from the rats for histopathological examinations.

### TUNEL assay

In order to visualize apoptotic neuronal death, paraffin sections (5 μm) of the brain tissues were assayed with the TUNEL reaction, using the Fluorescein FragEL DNA Fragmentation Detection kit (MERK, Germany). Briefly, paraffin sections were initially soaked in xylene, then deparaffinized by graded ethanol, and permeabilized in proteinase K [in 10 mM Tris (pH 8)]. The sections then were exposed to the TdT equilibration buffer, and the free 3′ hydroxyl groups of DNA were labeled with the fluorescein-conjugated deoxynucleotides. Nuclear staining was identified in cell nuclei with DAPI. The number of apoptotic nuclei and total number of nuclei in the injured area of cortex were counted in five standardized fields.

### Immunohistochemistry

Immunohistochemistry was performed to detect gliosis at 72 h after injury. Paraffin sections were initially soaked in xylene, then deparaffinized by graded ethanol, permeabilized with 3% Triton X-100 for 10 min, blocked with 10% normal donkey serum in PBS for 60 min at room temperature, and incubated overnight with primary antibodys at 4°C. Glial cells were labeled by rabbit anti-rat GFAP antibody (Cell Signaling Technology, Danvers, MA, USA; 1:800). Activated microglia was detected by rabbit anti-rat Iba-1 antibody (Wako Pure Chemical Industries Ltd., Osaka, Japan; 1:800). Primary antibodies were visualized using Alexa Fluor 488 donkey anti-rabbit secondary antibodies (Molecular Probes, Eugene, OR, USA; 1:500). GFAP and IBA1 positive cell in the peripheral area of injured cortex were counted in six standardized fields.

### Measurement of caspase-3 activity

The right side of cerebral cortex were dissected and homogenized in chilled phosphate buffered saline (PBS, 0.1M, pH 7.4), and then centrifuged at 10,000 *g* at 4°C for 10 min. The supernatants were collected, aliquoted, and stored at −80°C for following analysis. The tissue protein concentration was determined using a standard commercial kit (Beyotime Biological Technology Co., Ltd., Shanghai, PR China).

Caspase-3 activity of cerebral cortex was determined by using a Caspase-3/CPP32 Colorimetric Assay Kit (Beyotime Biological Technology Co., Ltd., Shanghai, PR China) under the manufacturer’s instructions. The absorbance of samples was measured by a microplate [enzyme linked immunosorbent assay (ELISA)] reader.

### Enzyme linked immunosorbent assay

The blood samples collected from left ventricule were stored at room temperature for 15 min to coagulate. The samples were centrifugalized for 20 min at the speed of 3000 rpm, and then the supernatant was removed carefully and preserved in −80°C for detection.

The serum and cerebral cortex levels of calcium binding protein β (S-100β), myelin basic protein (MBP), neuron-specific enolase (NSE), interleukin-8 (IL-8), interleukin-10 (IL-10), endothelin-1 (ET-1), inducible nitric oxide synthase (iNOS), hypoxia-inducible factor-1α (HIF-1α), and 3-nitrotyrosine (3-NT) were detected by standard ELISA kits (Yanxin Biological Technology Co., Ltd., Shanghai, PR China). The experiments were strictly performed under the manufacturer’s instructions. The absorbance of samples was measured by a microplate (ELISA) reader.

### Statistical analysis

All statistical analysis was performed using SPSS 19.0, a statistical software package. Data generated were expressed in the form of “means ± SD.” Statistical evaluation of the data was performed by univariate ANOVA (more than two groups) followed by Bonferroni’s multiple comparisons or unpaired t test (two groups). A value of *p* < 0.05 was considered statistically significance.

## Results

### Peak overpressure of the explosion blast wave and general mortality

**Figure 2:**
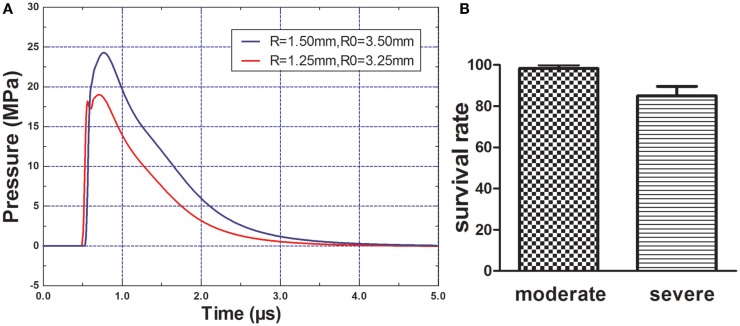
**Blast pressure wave and rats survival rate**. **(A)** Components and wave patterns of the blast pressure wave. R = radius of miniature spherical explosive; R0 = distance from scalp to the center of miniature spherical explosive. **(B)** Rats survival rate after blast-induced traumatic brain injury (bTBI), one rat (1/60) died in the moderate injury group and nine rates (9/60) died in the severe injury group.

The peak overpressure generated by the 2.5- and 3.0-mm diameter miniature spherical explosives was 19.01 and 24.27 Mpa, respectively (Figure 2A). After rats of each group were exposed to different intensity blasts, only one rat died in the moderate injury group at 1 h post-blast; within 3 h, nine rats died in the severe injury group (Figure 2B). No additional rats died in further study.

### Neurological scores

**Figure 3:**
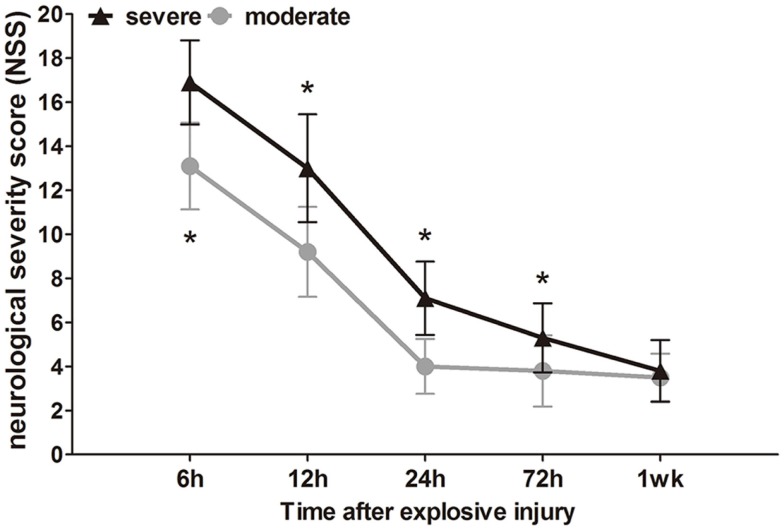
**Time course of NSS after blast-induced traumatic brain injury (bTBI)**. Sample numbers at each time points *N* = 20 (moderate = 10, severe = 10). Severe injury group vs. moderate injury group *p < 0.05.

The neurological impairment status of the injured rats was assessed by NSS at different times (6 h to 1 week) after explosion. Before bTBI, all rats attained a minimum score of 0. After explosion, the blast wave had directly impacted the non-dominant hemisphere (right), parietal cortex, and temporosphenoid lobe. Instantaneously, the blast wave was transferred to the brain tissue, and significant nerve damage symptoms were observed. High scores on motor, sensory, reflex, and beam-balance tests in the early phase (within 12 h) were observed, which demonstrated comparable neurological dysfunctions in both groups (*p* < 0.05). Animals in the severe group had consistent and significantly higher NSS values at early (within 12 h) and later time points (24 and 72 h) compared to the moderate injury group (*p* < 0.05), which indicated that the 3.0-mm diameter miniature spherical explosives could cause more severe damage than the 2.5-mm diameter miniature spherical explosives (Figure 3).

### Gross observation

**Figure 4:**
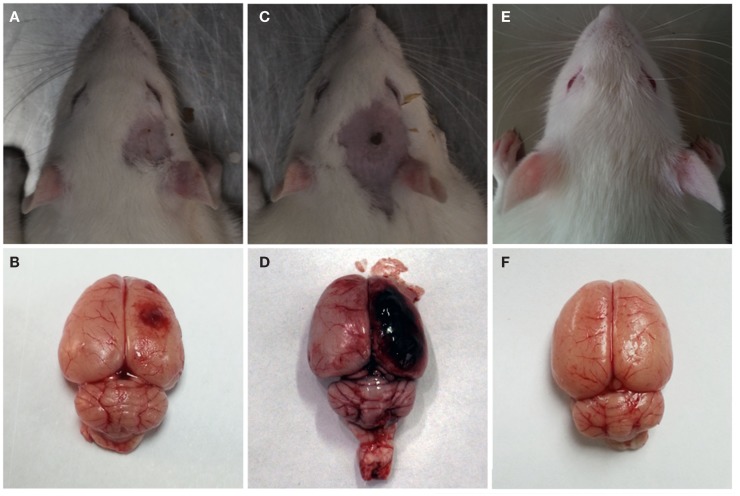
**Gross observation after blast-induced traumatic brain injury (bTBI)**. **(A,B)** Moderate injury group at 12 h after bTBI. **(C,D)** Severe injury group at 12 h after bTBI. **(E,F)** control group.

After the explosion, significant gross pathologic damage was visible in the two injury groups compared to the control group. The major pathological changes were visible capillary bleeds, cortical contusions, edemas, and extensive subarachnoid hemorrhages (Figure 4). The severe injury group had more serious subarachnoid hemorrhages and cortical contusions than the moderate injury group. Furthermore, no visible pathological damage was observed in the lungs, stomach, liver, kidney, or GI tract of any rats that underwent blast injury.

### Histopathological examination

**Figure 5:**
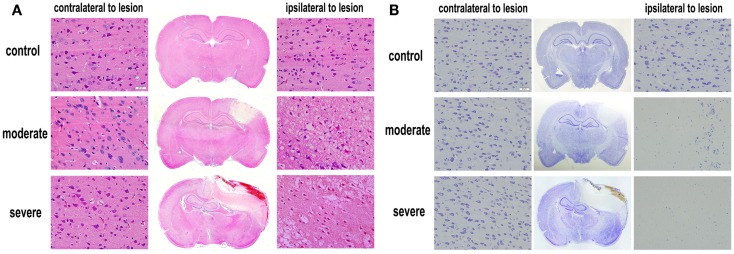
**Histopathological examination after blast-induced traumatic brain injury (bTBI)**. **(A)** HE-stained sections of brain tissues at 72 h after bTBI (scale bar = 20 μm). **(B)** Nissl-stained sections of brain tissue at 72 h after bTBI (scale bar = 20 μm).

Under the optical microscope, both HE-stained and Nissl-stained sections in the control group showed normal morphology and regular arrangement of neurons with clearly visible nuclei. However, in the injury groups, HE-stained and Nissl-stained sections showed cortical contusions and internal hemorrhages. The middle line of the brain shifted remarkably. In the cortex, cell loss area formed an apparent injured area (Figure 5). At 72 h after bTBI, the percentage of injured area to whole brain area was 7.78 ± 1.25% in moderate injury group and 18.37 ± 2.04% in severe injury group. The percentage of the severe injury group was significant larger than that of the moderate injury group (*p* < 0.05). HE-stained sections showed no visible damage on heart, spleen, kidney, lung, and liver at 6 h after bTBI (Figure S1 in Supplementary Material).

**Figure 6:**
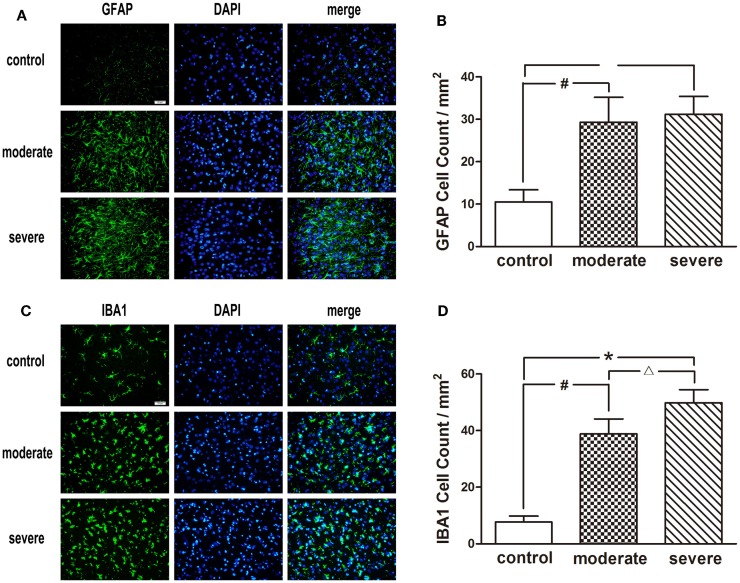
**Immunofluorescence examination in the peripheral area of injured cortex at 72 h after blast-induced traumatic brain injury (bTBI)**. **(A)** Astrocytes were stained by GFAP, the nuclei were stained by DAPI (scale bar = 20 μm). **(B)** Microglias were stained by IBA1, the nuclei were stained by DAPI (scale bar = 20 μm). **(C)** GFAP positive cell count in the peripheral area of injured cortex at 72 h after bTBI. **(D)** IBA1 positive cell count in the peripheral area of injured cortex at 72 h after bTBI.

After injury, astrocytes and microglia were densely distributed in the peripheral area of the injured cortex, which formed an obvious glial cell activation zone (Figure 6). Compared with the moderate injury group, a larger number of microglia were observed in the severe injury group. Furthermore, the increase of astrocyte branches and magnification of microglia bodies in the severe injury group was more significant than those in the moderate injury group.

### Caspase-3 activity and TUENL results

**Figure 7:**
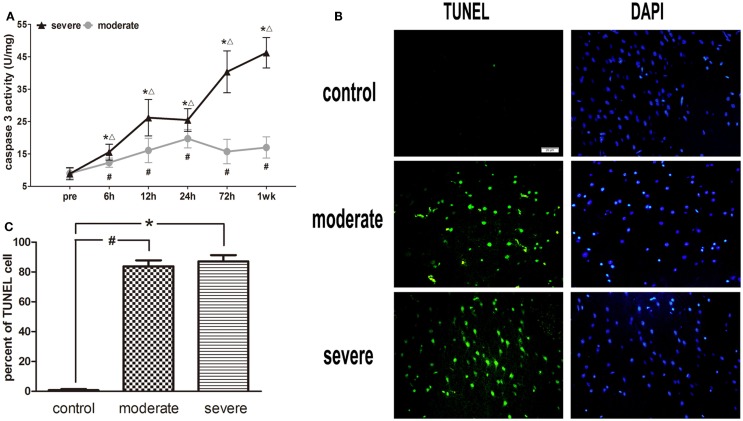
**Apoptotic cell death in brain tissue**. **(A)** Time course of caspase-3 activity after blast-induced traumatic brain injury (bTBI). Cortex sample numbers at each time points *N* = 12 (moderate = 6, severe = 6). Moderate injury group vs. control group ^#^*p* < 0.05; severe injury group vs. control group *p < 0.05; severe injury group vs. moderate group ^△^*p* < 0.05. **(B)** TUNEL staining in the injured area of cortex at 6 h after injury. **(C)** Percent of TUNEL cell in the injured area of cortex.

After injury, the activity of caspase-3 in the moderate injury group was elevated at 6 h, reached a peak at 24 h, and decreased at 72 h, while it was elevated again at 1 week. In the severe injury group, the activity of caspase-3 reached its first peak at 12 h, decreased slightly at 24 h, and then was significantly elevated until a second peak occurred at 1 week. At all post-injury time points, the activities of caspase-3 in the moderate and severe injury groups were significantly higher than those in the control group (*p* < 0.05). Statistical analysis found significant differences in the activity of caspase-3 between the severe injury group and the moderate injury group at 6, 12, 24, 72 h, and 1 week (*p* < 0.05) (Figure 7A).

Under the fluorescence microscope, rare TUNEL-positive cells were found in sections of the control group. The explosion markedly induced TUNEL-positive cell staining at 6 h post-injury, but the TUNEL-positive cells were limited to the injured area of cortex (Figure 7B). In addition, no significant difference was found in the apoptotic index between the moderate injury group and the severe injury group (Figure 7C).

### Expression of cellular damage markers (S-100β, MBP, and NSE)

**Figure 8:**
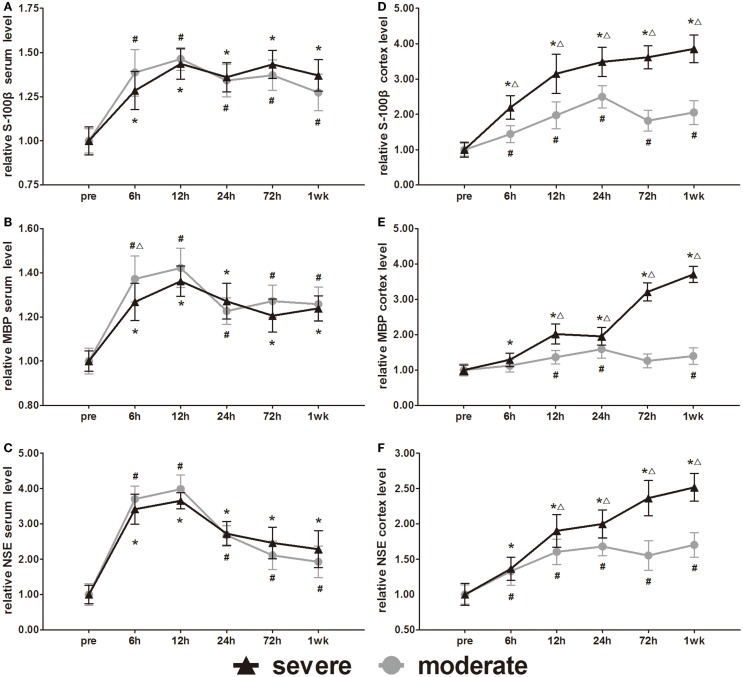
**Time course of level changes of biomarkers after blast-induced traumatic brain injury (bTBI)**. **(A)** Serum S-100β. **(B)** Serum MBP. **(C)** Serum NSE. **(D)** Cortex S-100β. **(E)** Cortex MBP. **(F)** Cortex NSE. Serum sample numbers at each time points *N* = 18 (moderate = 10, severe = 8); cortex sample numbers at each time points *N* = 12 (moderate = 6, severe = 6). Moderate injury group vs. control group ^#^*p* < 0.05; severe injury group vs. control group *p < 0.05; severe injury group vs. moderate group ^△^*p* < 0.05.

S-100β, MBP, and NSE are markers of glial and neuronal cell damage. Serum S-100β, MBP, and NSE increased significantly at 6 h compared to pre-injury levels and then peaked at 12 h. After 12 h, the MBP and NSE decreased slowly with time (Figures 8A–C). Following a decrease at 24 h, the S-100β showed a slight increase at 72 h. After injury, all serum levels of the three biomarkers were significantly higher than pre-injury levels (*p* < 0.05). Statistical analysis only found one significant difference in serum MBP at 6 h between the injury groups (*p* < 0.05). There was no significant difference found in serum S-100β and NSE between injury groups.

Cortex S-100β, MBP, and NSE of the moderate injury group reached the first peak at 24 h, decreased slightly at 72 h, and then reached a second peak at 1 week. Cortex S-100β, MBP, and NSE of the severe injury group maintained the trend of elevation at 1 week after injury (Figures 8D–F). Except for the cortex MBP levels of the moderate injury group at 6 and 72 h, all other cortex levels of the three biomarkers were significantly higher than pre-injury levels (*p* < 0.05). Cortex S-100β levels of the severe injury group were significantly higher than those of the moderate injury group at all post-injury time points (*p* < 0.05). The cortex MBP and NSE levels of the severe injury group were significantly higher than those of the moderate injury group at 12, 24, 72 h, and 1 week (*p* < 0.05).

### Expression of inflammatory cytokine markers (IL-8 and IL-10)

**Figure 9:**
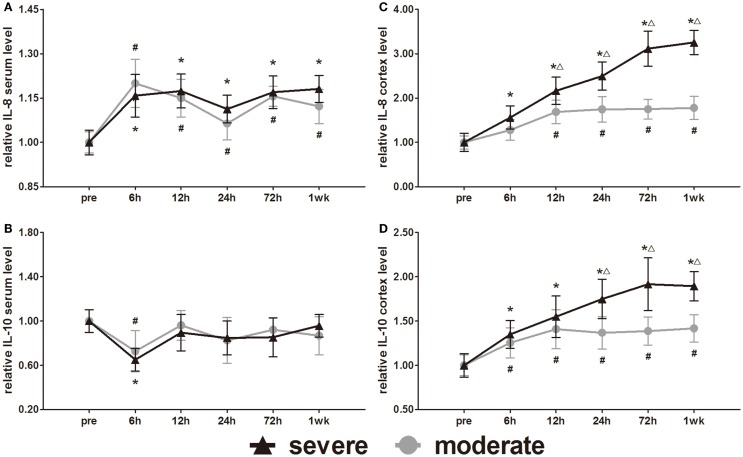
**Time course of level changes of biomarkers after blast-induced traumatic brain injury (bTBI)**. **(A)** Serum IL-8. **(B)** Serum IL-10. **(C)** Cortex IL-8. **(D)** Cortex IL-10. Serum sample numbers at each time points *N* = 18 (moderate = 10, severe = 8); cortex sample numbers at each time points *N* = 12 (moderate = 6, severe = 6). Moderate injury group vs. control group ^#^*p* < 0.05; severe injury group vs. control group *p < 0.05; severe injury group vs. moderate group ^△^*p* < 0.05.

IL-8 and IL-10 are markers of inflammation. Serum IL-8 and IL-10 showed opposite changes at 6 h (Figures 9A,B). Serum IL-8 showed a slight elevation, whereas serum IL-10 showed a slight decrease at 6 h. Subsequently, the serum IL-8 levels were maintained significantly higher than pre-injury levels (*p* < 0.05). After the initial decrease at 6 h, the serum IL-10 of both groups returned to pre-injury levels at all following time points. Statistical analysis found no significant difference in serum levels of IL-8 and IL-10 between the groups.

The IL-8 and IL-10 changes in the cortex were different from those in serum. After injury, the IL-8 and IL-10 were persistently elevated in both the moderate and severe injury groups (Figures 9C,D). Except for the cortex IL-8 levels of the moderate injury group at 6 h, all other cortex IL-8 and IL-10 levels were significantly higher than in the pre-injury group at all post-injury time points. Cortex IL-8 levels in the severe injury group were significantly higher than those in the moderate injury group at 12, 24, 72 h, and 1 week (*p* < 0.05). Cortex IL-10 levels of the severe injury group were significantly higher than those of the moderate injury group at 24, 72 h, and 1 week (*p* < 0.05).

### Expression of vascular constriction markers (ET-1)

**Figure 10:**
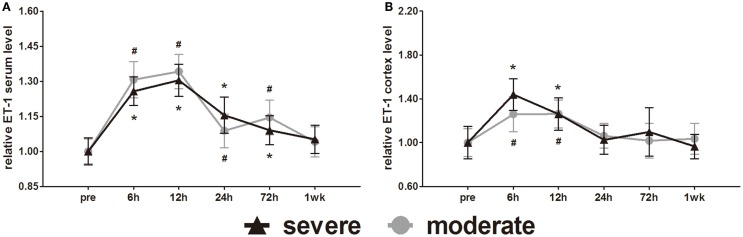
**Time course of level changes of biomarkers after blast-induced traumatic brain injury (bTBI)**. **(A)** Serum ET-1. **(B)** Cortex ET-1. Serum sample numbers at each time points *N* = 18 (moderate = 10, severe = 8); cortex sample numbers at each time points *N* = 12 (moderate = 6, severe = 6). Moderate injury group vs. control group ^#^*p* < 0.05; severe injury group vs. control group *p < 0.05; severe injury group vs. moderate group ^△^*p* < 0.05.

ET-1 is well known as a vasoconstrictor. Serum ET-1 was elevated at 6 h and reached a peak at 12 h. After 12 h, serum ET-1 decreased slowly with time (Figure 10A). Statistical analysis found no difference in serum ET-1 levels between injury groups. At 1 week post-injury, serum ET-1 levels of both groups were back to pre-injury levels. At 6, 12, 24, and 72 h post-injury, the serum ET-1 levels of both groups were significantly higher than pre-injury levels (*p* < 0.05).

Cortex ET-1 reached a peak at 6 h and then decreased with time (Figure 10B). Only at 6 and 12 h post-injury were the cortex ET-1 levels of both groups significantly higher than pre-injury levels (*p* < 0.05). Statistical analysis found no difference in cortex ET-1 levels between injury groups.

### Expression of metabolic change markers (iNOS, 3-NT, and HIF-1α)

**Figure 11:**
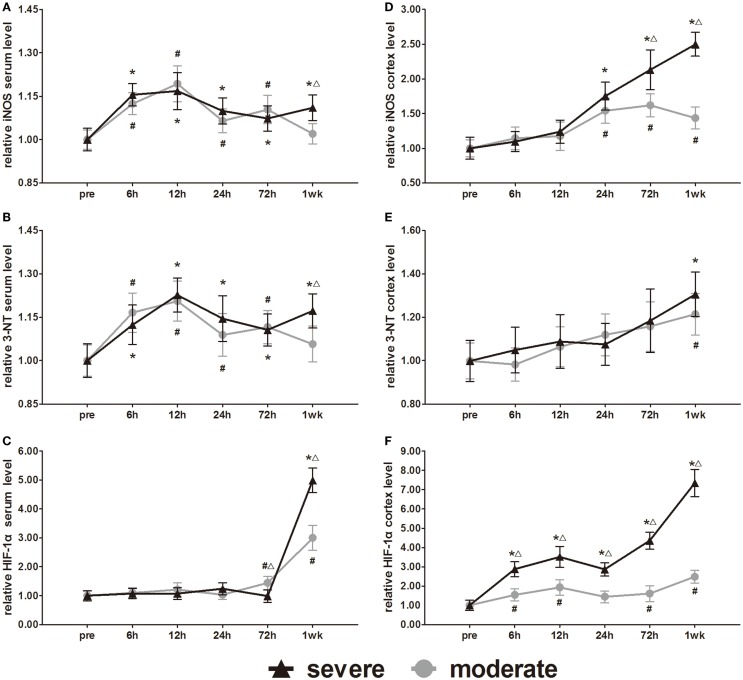
**Time course of level changes of biomarkers after blast-induced traumatic brain injury (bTBI)**. **(A)** Serum iNOS. **(B)** Serum 3-NT. **(C)** Serum HIF-1α. **(D)** Cortex iNOS. **(E)** Cortex 3-NT. **(F)** Cortex HIF-1α. Serum sample numbers at each time points *N* = 18 (moderate = 10, severe = 8); cortex sample numbers at each time points *N* = 12 (moderate = 6, severe = 6). Moderate injury group vs. control group ^#^*p* < 0.05; severe injury group vs. control group *p < 0.05; severe injury group vs. moderate group ^△^*p* < 0.05.

The metabolic markers iNOS, 3-NT, and HIF-1α were assessed in further experiments. Serum iNOS and 3-NT both showed biphasic responses to injury (Figures 11A,B). In the severe injury group, serum iNOS and 3-NT reached the first peak at 12 h and a second peak at 1 week. However, the onset time of the biphasic response in the moderate injury group was different from that in the severe injury group. In the moderate injury group, serum iNOS and 3-NT reached a first peak at 12 h and then a second peak at 72 h. At 1 week post-injury, serum iNOS and 3-NT levels of the moderate injury group recovered to pre-injury levels. Statistical analysis found significant differences in 3-NT and iNOS at 7 days between groups (*p* < 0.05). Changes of the serum HIF-1α levels were unique compared to the other markers (Figure 11C). Serum HIF-1α levels did not show any changes before 3 days, while after 3 days, the HIF-1α levels increased significantly in both injury groups. At 72 h and 1 week post-injury, serum HIF-1α showed significant differences between groups (*p* < 0.05).

All the iNOS, 3-NT, and HIF-1α changes in the cortex were significantly different from those of serum. After injury, all cortex levels of the three markers showed gradually increasing trends (Figures 11D–F). At 24, 72 h, and 1 week post-injury, cortex iNOS levels of the severe injury group were significantly higher than those of the moderate injury group (*p* < 0.05). Only at 1 week, post-injury was the cortex 3-NT levels of both groups significantly higher than pre-injury levels (*p* < 0.05). Statistical analysis found no significant difference in the cortex levels of 3-NT between groups. At all post-injury time points, cortex HIF-1α levels of the severe injury group were significantly higher than those of the moderate injury group (*p* < 0.05).

## Discussion

Accompanying with the rapid development of military technology, bTBI caused by explosive devices has become the predominant cause of casualties in modern warfare and civilian terrorism. When an explosive device detonates, high-pressure gasses rapidly expand from its point of formation, compressing the surrounding air or water and generating a pressure pulse with explosive energy. The resulting “blast wave” propagates in all directions (Oran and Williams, 2012). The blast wave can cause injury to multiple organs, and the brain is one of the most vulnerable organs to damage by such a blast (Finkel, 2006; Bhattacharjee, 2008; Ritenour and Baskin, 2008). Even if the parameters of the blast wave are the same, due to the heterogeneous contents of brain tissue and different propagation speeds of the blast wave in various media, the etiology of bTBI is complicated (Wightman and Gladish, 2001; Chen et al., 2009). Although clinical data from patients provide some clues for investigating the mechanism of bTBI, the individual variation of patients leads to a lack of reliable conclusions. Therefore, over the past several decades, many researchers tried to collect repeatable data by establishing animal bTBI models.

Currently, various types of experimental animal models have been used to investigate the mechanisms of bTBI, including blast tubes with explosives, shock tubes with compressed air or gas, open field exposure, and animals fixed in cabin models (Saljo et al., 2000; Wang et al., 2003; Long et al., 2009; Cheng et al., 2010; Alley et al., 2011; Cernak et al., 2011; de Lanerolle et al., 2011; Koliatsos et al., 2011; Kuehn et al., 2011; Rubovitch et al., 2011). However, these animal models have significant problems. First, when the entire animal body is fixed in a tube or cabin and exposed to a blast wave, there is insufficient protection to the body. The blast wave can pass through the simple protection and cause damage to multiple organs. Second, precise control of the brain injury position is not satisfactory. There is no doubt injury to different areas of the cerebrum, cerebellum, or brain stem will produce different consequence. Third, an electric detonator with a metal or paper shell will produce fragments when it is detonated that may penetrate the scalp or skull and cause additional injury.

To solve these problems, we established a novel bTBI model in rats, which uses a miniature explosive to create a realistic blast wave. All miniature explosives composed of PETN were shaped into 2.5 or 3.0 mm diameter spheres and were produced in accordance with constant parameters, which ensured that a consistent explosion force and homogeneous blast wave propagated in all directions. The usage of miniature explosives minimized the influence of the blast on other organs. By using an electric detonator and a silver fuse to ignite the miniature explosives, fragments from the detonator could be shielded by the metal sleeve, which was located in the center of the specially designed apparatus. The distance and position of the miniature explosives relative to the animals could be precisely adjusted by a knob on the apparatus. In our previous experiments, we controlled the distance between the 2.5-mm diameter spherical explosive and the scalps of the rats from 1 to 4 mm to evaluate the degree of damage. The blast-wave pressure sharply decreased as the distance increased. Survival rate at 1 mm was 20% (*n* = 20), and there was no obvious damages to the brain tissue when the distance was ≥4 mm. In this study, the explosives were fixed at 2 mm over the scalp and 3 mm right of the center point between the bregma and lambdoid suture. Another advantage of this apparatus was its ability to create eight bTBI rats at one time using a single electric detonator. After the blast, general morphological examination and histological evidence showed that the severe group had more serious injuries than the moderate group. Gliosis and microglia activation were observed in the areas surrounding the lesions, which were proportionate to the intensity of the explosion. These pathological changes at the cellular level were similar to the brain trauma induced by the controlled cortex impact (CCI) model. All these data confirmed the reality, repeatability, and controllability of our novel animal model to simulate bTBI.

In traumatic brain injury (TBI), a lot of biomarkers have showed important roles in diagnosing the disease, monitoring progress, predicting outcome, and providing pertinent molecular information about ongoing pathological changes for designing evidence-based therapeutic interventions (Kochanek et al., 2011). On the basis of this novel bTBI model, we investigated the changes of various biomarkers in the serum and cortex. S-100β, MBP, and NSE were considered to be biomarkers of neuronal and glial cell death (Agoston and Elsayed, 2012). Some studies showed that serum S-100β, MBP, and NSE could correlated well with the injury magnitude and outcome in TBI (Neher et al., 2014). However, our study showed that the levels of S-100β, MBP, and NSE in serum did not match well to those in the cortex, nor did they correlate with the extent of bTBI. Similar results were also observed in other study based on swine bTBI model (Gyorgy et al., 2011). The reason for the difference between TBI results and bTBI results might be that the integrity of the blood–brain barrier was not completely destroyed by the blast wave. Inflammation is almost always a result of injury, and occurs in response to damaging stimuli, triggering the release and activation of cytokines and chemokines and the activation and proliferation of microglia (and astroglia) in the CNS (Agoston and Elsayed, 2012). After bTBI, the proliferation of microglia and astroglia and the high expression of IL-8 and IL-10 in the cortex were both observed in our study. The increases of other inflammation cytokines such as IL-1α, IL-6, and TNF-α were also confirmed in other bTBI studies (Dalle Lucca et al., 2012; Cho et al., 2013). All these results implied that anti-inflammation therapy in the acute phase may help to attenuate bTBI. Vasospasm was thought to be an important pathological change after TBI (Salonia et al., 2010; Agoston and Elsayed, 2012). ET-1 as a powerful vasoconstrictor has been reported would significantly elevate in the cerebrospinal fluid of patients with head injury (Salonia et al., 2010). However, the expression of ET-1 has never been determined in a bTBI model. We found that the significant increase of cortex ET-1 was only in the acute phase of bTBI, but the significant increase of serum ET-1 would persist a relative long period. The increase of systemic ET-1 might be one type of traumatic stress response, which would help to keep cerebral perfusion. Metabolic disorders, including hypoxia and oxidative stress, were also assessed in this study. Our results of iNOS and 3-NT showed that systemic oxidative stress was prior to brain oxidative stress, and the brain oxidative stress mainly occurred in the later phase after bTBI. However, some mild bTBI studies showed that in the acute phase of bTBI, remarkable oxidative stress had existed in the brain (Readnower et al., 2010; Abdul-Muneer et al., 2013). Agoston and Elsayed (2012) have proposed a notion that the onset, intensity, and temporal patterns of various bTBI pathological processes likely depend on the severity of the injury. The differences in the results of oxidative stress may because the bTBI in our study was more severe. Studies have proved that HIF-1α plays a important role in neuronal apoptosis, blood–brain barrier disruption, and brain edema after TBI (Higashida et al., 2011; Li et al., 2013). However, the expression of HIF-1α after bTBI has never been studies. Our result showed that in the acute phase of bTBI, the HIF-1α level had significantly elevated in brain. Thus, active prevention of hypoxia should be incorporated in treatment strategies for bTBI.

In conclusion, we established a novel bTBI model in rats using miniature explosives. Gross pathology, histopathological variability, neurological functions, and expressions of various biomarkers were examined after explosive injury, indicating the satisfactory repeatability and controllability of this novel animal model. This novel bTBI model can provide a reliable experimental platform to investigate the mechanism underlying bTBI and facilitate the development of treatment strategies for patients undergoing blast injury.

## Conflict of Interest Statement

The authors declare that the research was conducted in the absence of any commercial or financial relationships that could be construed as a potential conflict of interest.

## Supplementary Material

The Supplementary Material for this article can be found online at http://journal.frontiersin.org/article/10.3389/fncel.2015.00168
