# Supplementary material for: A Novel Rat Model of Blast-Induced Traumatic Brain Injury Simulating Different Damage Degree: Implications for Morphological, Neurological, and Biomarker Changes
Source: Front Cell Neurosci. 2015 May 1;9:168. doi: 10.3389/fncel.2015.00168 (PMC4416450; doi:10.3389/fncel.2015.00168)

Fig. S1. HE-stained sections of liver, lung, kidney, spleen and heart at 6h after blast-induced traumatic brain injury (bTBI).

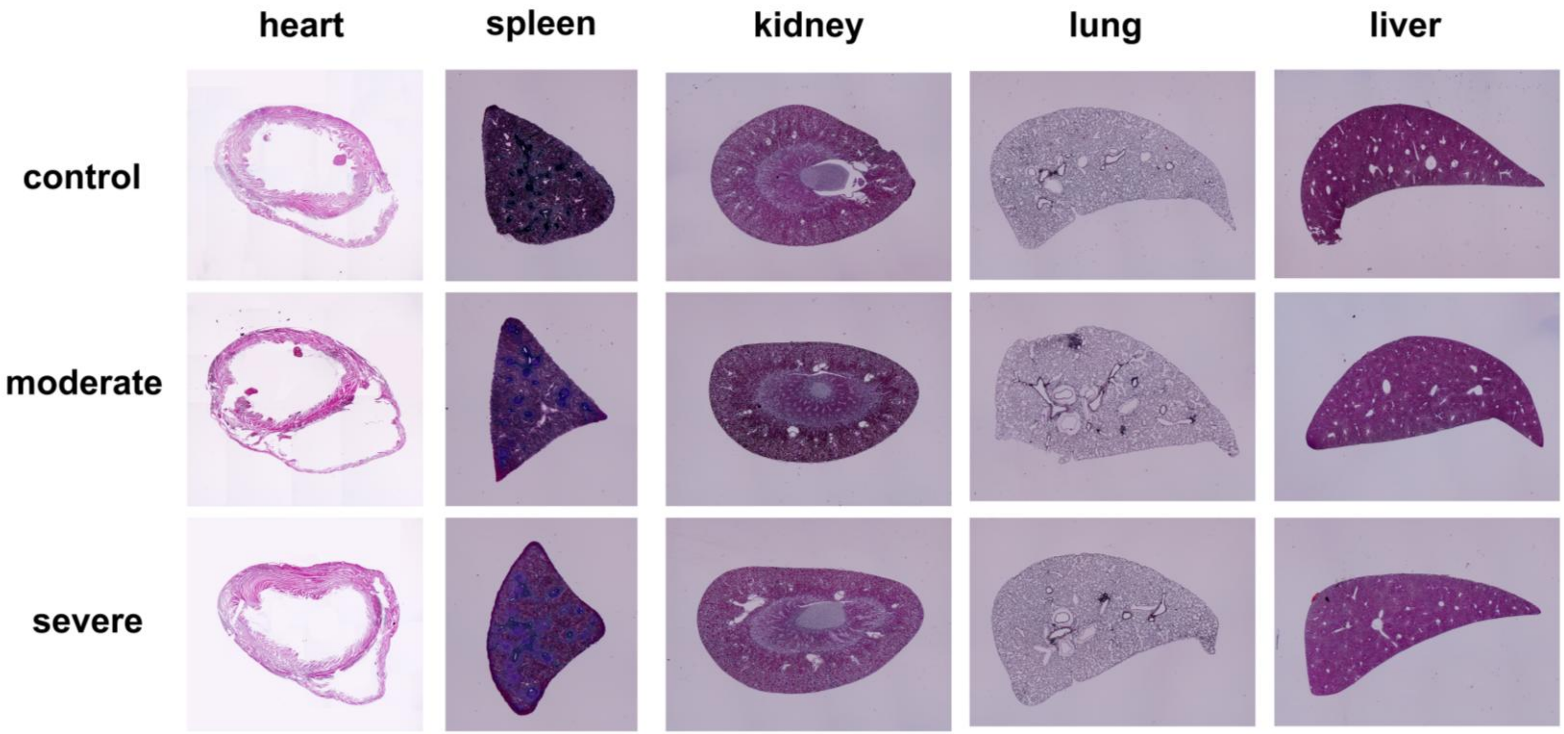

Supplement: Supplementary file 2 [file Image_1.PDF]
